# Mechanical and Surface Properties of Resilient Denture Liners Modified with Chitosan Salts

**DOI:** 10.3390/ma12213518

**Published:** 2019-10-26

**Authors:** Maike Herla, Klaus Boening, Heike Meissner, Katarzyna Walczak

**Affiliations:** Department of Prosthetic Dentistry, Carl Gustav Carus Faculty of Medicine, Technische Universität Dresden, Fetscherstr. 74, 01307 Dresden, Germany; maike.herla@mailbox.tu-dresden.de (M.H.); Klaus.Boening@ukdd.de (K.B.); Heike.Meissner@ukdd.de (H.M.)

**Keywords:** chitosan, chitosan HCl, chitosan glutamate, resilient denture liner, Shore A hardness, surface roughness

## Abstract

Chitosan (CS) and its derivatives show antibacterial and antifungal properties and could help treat and prevent denture stomatitis (DS). Mechanical and surface properties of resilient denture liners were evaluated when modified with CS salts. CS-hydrochloride (CS-HCl) and CS-glutamate (CS-G) were added to resilient denture liners Ufi Gel P and Coe-Soft at four different concentrations (0.1%, 0.2%, 0.4%, 1% w/w) from which specimens were produced, as well as a control group of each material with no added CS salt. Ten specimens per group (Ø 35 mm, height 6 mm) were manufactured. They were stored in distilled water at 37 °C for a total of 30 days (d). Shore A hardness (SHA) and surface roughness (Ra) were evaluated after 24 h (T1), 7 d (T2), 14 d (T3) and 30 d (T4). Kruskal–Wallis and U-test (Bonferroni-Holm adjusted) were used for statistical analysis (*p* ≤ 0.05). Ra increased significantly once CS salts were added. SHA increased significantly for some groups, but all specimens fulfilled requirements set by ISO 10139-2:2016. Modification with CS salts does not influence the mechanical properties of the modified resilient denture liners in a clinically relevant manner. Despite the increased roughness, the concept is suitable for further studies. Especially antimicrobial/antibiofilm studies are needed.

## 1. Introduction

Denture stomatitis (DS) is a disorder specified by inflammation of oral tissues covered by dentures and can affect over 70% of denture wearers [1]. Infection with *Candida albicans* is considered to be the predominant factor for developing DS [2], often supported by poor denture hygiene or trauma caused by ill-fitting dentures or continuous wear [1,2].

*C. albicans* is a yeast [3] and a commensal in the oral cavity, which can adhere to host cells, other *C. albicans* cells, other microorganisms [4] and to abiotic surfaces like dentures and form biofilms there [4], which can lead either to topical [5] or systemic infections [4]. Biofilm formation makes *C. albicans* more resistant towards antimicrobial agents compared with planktonic cells [6]. Furthermore, *Candida* biofilms can survive high concentrations of commonly used antifungals such as azoles [7]. Treatment of DS lacks a gold standard therapy [8]. DS can be treated, e.g., by local or systemic antifungal therapy, denture disinfection and cleansing or laser treatment of affected tissue [8]. When treating DS with antifungal drugs, though symptoms did improve, recolonization of the oral tissue could not be prevented [2], favouring recurrence of DS. Furthermore, side effects like drug-resistant fungi and the toxicity of existing drugs [9] are associated with the treatment of *C. albicans.* Patients often experience no discomfort when suffering from DS which can lower patient compliance [10]. Unpleasant taste and the need for frequent use might be further restricting factors [11]. Therefore, the establishment of natural antimicrobial agents is explored [9], considering they are affordable, commonly available and have fewer side effects than synthetic antifungals [11]. As ill-fitting dentures can also promote DS, resilient denture liners can be a long-term solution, providing a cushion-like effect to traumatized oral mucosa [12].

Resilient denture liners are layered between oral mucosa and denture base [13]. They are silicone elastomers or plasticized acrylic resins [13], which can either be auto-polymerized or heat-cured [14]. Furthermore, there is a differentiation between short- and long-term resilient denture liners [15]. Whilst long-term denture liners have to keep their resilience for 30 days and can be used for up to 12 months [15], short-term denture liners are required to keep their mechanical properties for 7 days [16] and are made to use for a maximum of 30 days [15]. The present study focuses on long-term resilient denture liners, investigating the modification of an autopolymerising silicone resilient denture liner and one based on plasticized acrylic. They are indicated in elderly patients with atrophied or sharp alveolar ridges, thin oral mucosa that does not tolerate the pressure caused by the dentures, patients suffering from pain at nerve exit points or reoccurring pressure marks [14]. 

Colonization with *C. albicans* on and within the resilient denture liner [14,17], favouring DS, is a major disadvantage of these materials. In vivo, microorganisms on soft lining materials can already be detected within the first day and colonisation increases over time [18,19]. In conclusion, eradicating the microorganisms from and preventing biofilm formation on and within the resilient denture liner seems favourable in order to prevent and treat DS. Adding different antimicrobial drugs but also natural antimicrobial agents like silver nanoparticles, tea tree oil or lemongrass essential oil to temporary soft liners (tissue conditioners) and resilient denture liners, mostly in vitro, has been explored over the years [11]. However, to the authors’ knowledge there is still no established antimicrobial/antibiofilm modification of resilient denture liners that could prevent DS or frequent relining due to microorganism colonisation or deterioration of the liner, which can be a burden for elderly patients. Novel approaches to develop a widely accepted solution are therefore still necessary.

However, additives can change material properties such as hardness or surface roughness [20,21,22]. Both are important in the clinical setting and standards e.g. declared by ISO ensure safe clinical utilization. 

The Shore A hardness (SHA) test is non-destructive and can be used e.g. for resilient denture liners. It measures the reaction force upon the indentation of the material tested [23]. 

Surface roughness can be measured e.g. by the arithmetic mean deviation of a surface from the average height (Ra) [24]. Surface roughness influences microorganisms’ initial adherence and rougher surfaces tend to be more colonized by yeasts [12,17,25]. For dentures, 0.2 µm is considered a threshold in surface roughness below which reduction of biofilm accumulation can no further be expected [26]. 

CS is a linear biopolymer [27] obtained when chitin is partially deacetylated in alkaline conditions [28]. It is biocompatible, antimicrobial, has regenerative properties and is commonly available [29]. CS has amino and hydroxyl groups and acts polycationic when its amine groups are protonated at low pH [27]. It can be solved in acidic solutions of pH < 6 [30] and its salts are soluble in water [31,32]. Antibacterial and antifungal properties of CS and its derivatives are described in multiple papers and reviews [28,31,33,34] and depend, e.g., on its molecular weight (MW), degree of deacetylation (DDA) as well as the type of microorganism [34]. Most antimicrobial actions are due to its ability to interact with negatively charged cell components such as lipopolysaccharides of Gram-negative bacteria [35], lipoteichoic acids of Gram-positive bacteria [36] or fungi’s phospholipid bilayer in their cell membranes [34]. CS was shown to inhibit *C. albicans* from forming a normally structured biofilm in vitro, and CS-treated biofilms grew slower compared with untreated ones [37]. CS salts (e.g. CS-HCl, CS-G) showed antimicrobial activity against *C. albicans* or Gram-positive and Gram-negative bacteria [38,39]. Because of the previously described activities of CS, CS salts and further CS derivatives, they seem to be promising contenders for creating antimicrobial denture materials, especially resilient denture liners. 

Modifications of tissue conditioners with CS, CS nanoparticles, quaternized CS and CS-oligosaccharide are described [40,41,42]. These studies show promising antifungal activity of modified materials but do not evaluate mechanical properties. To the best of the authors’ knowledge, only studies describing modifications of resilient denture liners with other particles (silver nanoparticles; nystatin, miconazole, ketoconazole, itraconazole, chlorhexidine) evaluated mechanical properties [20,21,43]. Furthermore, no studies were found describing the addition or modification of resilient denture liners with CS salts such as CS-G and CS-HCl. 

In a previous pilot project, the authors investigated the flexural strength of a hard denture base material modified with CS powder (MW 200–400 kDA, DDA 87.6% to 92.5%, unpublished data), which decreased drastically. Thus, the modified material was not suited for use as a denture base material. The reason for the decreased flexural strength probably was the large grain size of CS-powder used. CS-HCl and CS-G are available in smaller grain size. Therefore, they might influence the mechanical properties of modified dental materials less and could be promising for developing antifungal materials for the prevention and treatment of DS.

The aim of this study was to evaluate Shore A hardness (SHA) and surface roughness (Ra) as mechanical and surface properties of two resilient denture liners modified with CS salts as possible antimicrobial agents for treating and preventing DS. For SHA this could be assessed by evaluating whether CS salt modified resilient denture liners still fulfilled ISO 10139-2:2016 requirements and showed clinically acceptable values. For Ra, values were compared over time and in relation to 0.2 µm as stated above. 

The following null hypotheses were stated:

- SHA in CS salt-modified (CS-HCl, CS-G) resilient denture liners do not differ from unmodified material (control group) at different points in time;
- Ra in CS salt-modified (CS-HCl, CS-G) resilient denture liners do not differ from unmodified material (control group) at different points in time.

## 2. Materials and Methods

### 2.1. Specimen Preparation

**Figure 1:**
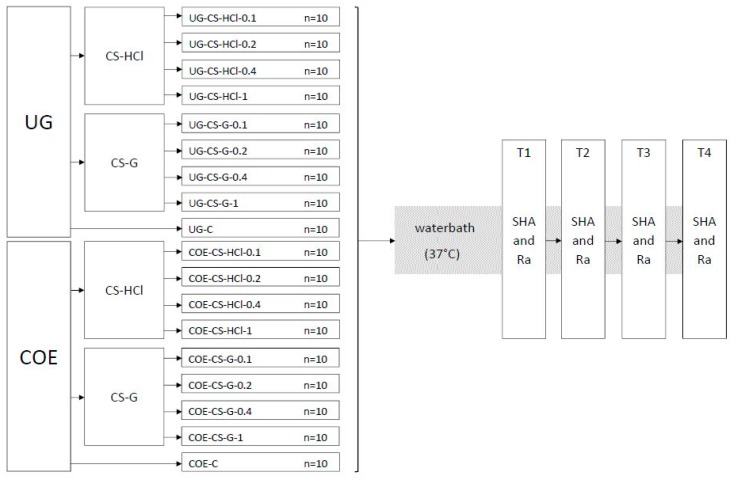
Flowchart of the experiment groups and testing procedure. Ufi Gel P (UG), Coe-Soft (COE), chitosan hydrochloride (CS-HCl), chitosan glutamate (CS-G), control group (C), concentration in % w/w of CS salt added (0.1, 0.2, 0.4, 1), Shore A hardness (SHA), surface roughness (Ra), 24 h after specimen preparation (**T1**), 7 d after specimen preparation (**T2**), 14 d after specimen preparation (**T3**), 30 d after specimen preparation (**T4**).

Eighteen groups of specimens (each group n = 10) were produced and the experiments were conducted as shown in the flowchart (Figure 1). 

**Figure 2:**
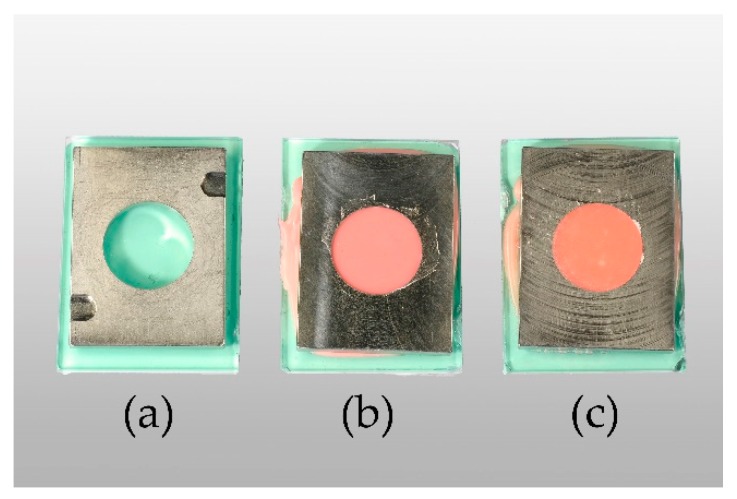
From left to right: empty templet (**a**), Ufi Gel P (UG) specimen in templet after setting (**b**), Coe-Soft (COE) specimen in templet after setting (**c**).

In accordance with ISO 10139-2:2016, specimens measured 35 mm in diameter and 6 mm in height [44]. Ufi Gel P (UG, VOCO GmbH, Cuxhaven, Germany) and Coe-Soft (COE, GC America Inc., Alsip, IL, USA) were used as resilient denture liners. Chitosan hydrochloride (CS-HCl) and chitosan glutamate, both commercially purchased from Heppe Medical Chitosan GmbH (Halle, Germany), were used as CS salts. DDA and MW measurements were done by the manufacturer by the use of gel permeation chromatography (GPC) for MW determination. DDA was determined according to Ph. Eur. 9.0, 1774 (01/2017) [45]. Specifications of the products given by the manufacturer are shown in Table A1. A templet made from stainless steel was used to produce standardised specimens (Figure 2a–c). In order to attain smooth surfaces, glass plates were placed on the top and bottom of the templet. To stabilize and keep the glass from breaking, silicone (Provil novo Putty, Kulzer GmbH, Hanau, Germany) and a metal plate were added to the glass plates’ lower surface.

**Figure 3:**
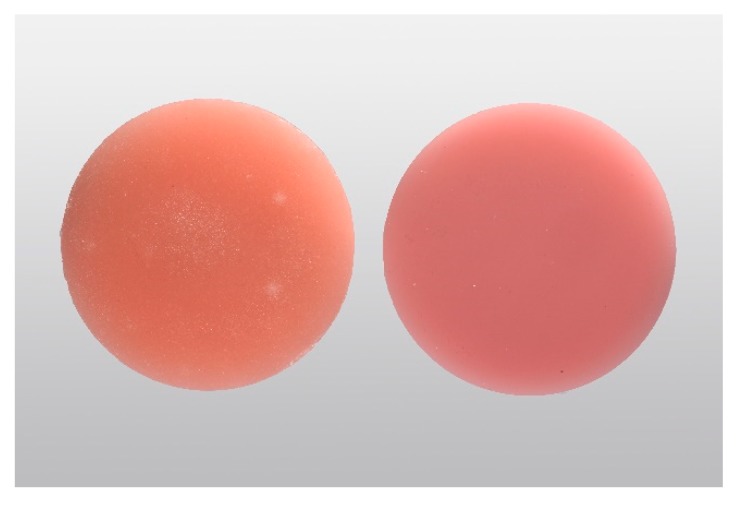
From left to right: Coe-Soft (**COE**) specimen after setting, Ufi Gel P (**UG**) specimen after setting.

Both resilient denture liners were mixed according to the manufacturer’s instruction. UG base and catalyst (4.5 g of each) were used for one specimen and 5.5 g of powder and 4 ml of liquid for COE, respectively. CS salt powders in concentrations 0.1%, 0.2%, 0.4% or 1% w/w were added to UG pastes and COE liquid, mixing well before adding the powder. For the specimens in the control groups, no CS salts were added (Figure 3).

The components were combined until homogenous and placed into the templet. They were then put into a hydraulic flask press (Type 5414, KaVo Elektrotechnisches Werk GmbH, Leutkirch im Allgäu, Germany) for 20 s before being clamped together and, deviating from ISO 10139-2:2016 [44], moved into an incubator (Heraeus B 6030, Thermo Fisher Scientific, Waltham, MA, USA) at 37 °C for 10 min to set, which is supposed to mimic the temperature of the oral cavity. Specimens were removed from the templets and stored in distilled water at 37 °C for a total of 30 d (Thermocycler THE-1100, SD Mechatronik GmbH, Feldkirchen-Westerham, Germany) in accordance to ISO 10139-2:2016. They were only taken out of the water during the time of SHA and Ra testing. 

### 2.2. Shore A Hardness

**Figure 4:**
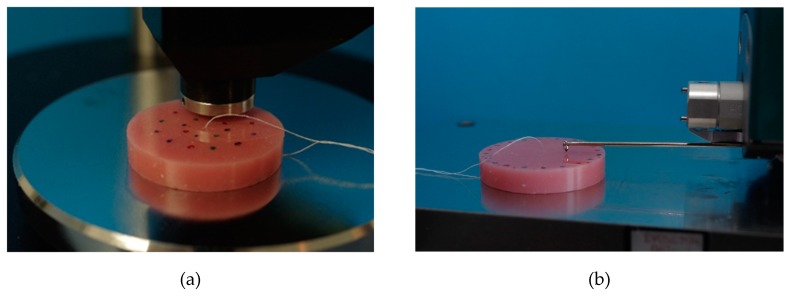
Shore A hardness testing with a digital durometer (**a**), surface roughness testing with contact profilometry (**b**).

SHA was tested according to ISO 10139-2:2016 at different points in time: 24 h (T1) and 30 d (T4) and additionally at 7 d (T2) and 14 d (T3). SHA tests were performed accurate to ± one hour at T1–T4. On one side of the specimens, SHA was measured at 5 measurement points with a digital durometer (HDD-2, Hildebrand Prüf - und Meßtechnik GmbH, Oberboihingen, Germany; operating stand OS-2, Rex Deutschland Hildebrand GbR, Oberboihingen, Germany) after a 5 s impact, resulting in possible SHA values from 0 to 100 [46]. A standardized templet was used to ensure the position and fulfil the minimum distance between the measuring points as well as ensuring new measurement positions at T1–T4. The measurement points were distributed evenly with at least 2 mm in between two points and a 5 mm distance from the specimen’s boundary (Figure 4a). For each point in time, a SHA mean value per specimen was calculated from the 5 measurement points (Excel 2016, Microsoft Office, Microsoft Corporation, Redmond, WA, USA).

### 2.3. Surface Roughness

Surface roughness was tested after SHA at the same 4 points in time T1, T2, T3 and T4, accurate to ± one hour each time. Roughness measurement was performed by contact profilometry with hommel etamic W20 (Jenoptik Industrial Metrology Germany GmbH, Villingen-Schwenningen, Germany) at 5 measurement points on the opposite side to SHA testing of each specimen. To ensure the position of measuring points at each point in time (T1–T4) and to not double the use of one measuring point, a standardized templet was used. Cut off was set at 0.8 mm, the total measured length was 4.8 mm. The specimens were placed in the profilometer, the linear traverse unit was positioned parallel to the specimen’s surface, then aligned and adjusted (Figure 4b). Ra parameter (arithmetic mean roughness) was evaluated. For each point in time and for each specimen, the mean values of Ra were calculated (Excel 2016, Microsoft Office, Microsoft Corporation, Redmond, WA, USA). 

### 2.4. Statistical Analysis

Mean values of SHA and Ra per group and point in time were used for further statistical analysis. Shapiro–Wilk test was used to test for normal distribution. Kruskal–Wallis test and U-Test were performed using SPSS software (IMB SPSS 25, IMB, Armonk, NY, USA) for Windows. Bonferroni–Holm correction was used to adjust *p*-values. The level of significance was set at α = 0.05.

## 3. Results

As not all groups were distributed normally, non-parametric tests were chosen for further statistical examination.

### 3.1. Shore A hardness

For both resilient denture liners (UG and COE), SHA values increased over the 30-day trial compared to 24 h (Table 1). For UG, SHA values were highest at 14 d whilst for COE they were mostly highest at 30 d. The UG control group (UG-C) showed mostly the lowest median SHA values at all points in time (T1, T2, T3, T4). Other than UG, at 30 d COE control group (COE-C) showed the highest SHA values.

SHA values of UG modified with CS-HCl in concentrations of 0.1% at 24 h and 30 d or with 0.2% at 24 h or 1% at 30 d did not differ significantly from their respective control group. Moreover, UG modified with 0.1% CS-G at 24 h showed no significant differences to the control group. All other UG groups differed significantly compared to UG control group (Table 2).

SHA values for COE modified with CS-HCl in concentrations of 0.2% at 30 d or with 0.4% and 1% at 14 d and 30 d showed significant differences compared to the control group (*p*-value raw and adj.) Regarding COE modified with CS-G, significant differences were found for 0.2% at 14 d (raw *p*-value only) and 0.2%, 0.4% and 1% at 30 d (*p*-value raw and adj.). All other modified COE groups showed no significant differences compared to the COE control group (Table 3).

Still, all groups at 24 h and 30 d showed mean SHA values that fulfilled ISO 10139-2:2016 requirements. Furthermore, all single SHA values acquired as raw data fulfilled the ISO standard. SHA values at 7 d and 14 d also met conditions set by the norm, despite ISO 10139-2:2016 not requiring measurements at the 7 d or 14 d time stamp (for mean SHA values see Table A2).

### 3.2. Surface Roughness

Surface roughness (Ra) increased when CS salts were added to resilient denture liners (Table 4). 

For UG groups the lowest median Ra value was found in the UG control group at 24 h whilst the highest was found in UG modified with CS-HCl in a concentration of 1% at 30 d. Ra values of all UG groups that contained CS salt were significantly higher (*p* < 0.001 raw and adj. for all groups) compared with control group at all registered points in time (T1, T2, T3, T4). Ra values for UG generally became higher, the higher the concentration of CS salts added and the more time had passed. For COE groups the lowest median Ra value was found in the COE control group at 30 d whilst the highest was found in COE modified with CS-G in a concentration of 1% at 7 d. Ra values of all COE groups at all registered points in time (T1, T2, T3, T4) apart from COE modified with CS-G in a concentration of 0.1% at 24 h differed significantly from unmodified material in the COE control group (Table 5).

At 30 d, the higher the concentration of CS salt added to COE, the rougher the specimen was, apart from COE modified with CS-G in a concentration of 0.1%, which was slightly rougher than COE modified with CS-G in a concentration of 0.2% (median). Especially for COE-C and COE groups modified with CS salts at lower concentrations (0.1% and 0.2%) specimens became less rough with time. 

Ra values lower than 0.2 µm should be considered ideal for reasons stated above. In this study not even control groups UG-C and COE-C, which were less rough than modified groups, were able to achieve mean or median Ra values ≤0.2 µm, apart from median value for UG control group at 24 h (for mean and SD values of Ra see Table A3). Although the UG control group at 24 h, 14 d and 30 d did show minimal Ra values ≤0.2 µm, median values at 14 d and 30 d as well as maxima in all three groups were higher than 0.2 µm. 

Apart from UG modified with CS-HCl in a concentration of 1%, all UG groups were less rough than the least rough COE group (COE control group at 30 d). Therefore, even modified UG groups showed more favourable Ra values than COE.

## 4. Discussion

The influence of modification of long-term resilient denture liners with CS salts on their mechanical and surface properties (SHA and Ra) was investigated. According to the manufacturers, relining with UG lasts between 2 weeks and 2 years, whilst COE lasts about 3 months. These materials are therefore considered long-term resilient denture liners by the authors and have both already been modified with antimicrobial particles different than CS-salts in other studies [21,47].

The null hypothesis for SHA must be accepted partly. The null hypothesis for Ra must only be accepted for COE modified with CS-G in a concentration of 0.1% at 24 h. 

Despite all groups fulfilling ISO 10139-2:2016 requirements at all registered points in time regarding SHA values, changes in surface roughness seem to worsen material properties in relation to the postulated value of 0.2 µm. 

Ideally, resilient denture liners are supposed to increase patients’ comfort by helping distribute functional stress evenly [14]. An increase in hardness is undesirable, reducing the absorbing effect [14,48]. It was reported that acrylic-based resilient denture liners hardened more within 6 months than silicone resilient denture liners [14]. On the contrary, another study investigating hardness values over a total of 28 days showed less hardness increase for an acrylic-based resilient denture liner than for a silicone one [14]. The latter could not be confirmed in the present study. Overall, the acrylic-based resilient denture liner showed greater changes in SHA than the silicone-based one after 30 days, which was also concluded by other authors [49]. Modifications of an acrylic-based resilient denture liner (COE) with CS salts showed less of an increase in hardness than the control group, whilst the contrary was shown for the silicone resilient denture liner (UG). This could be because of how these materials are composed. Plasticizers ensure initial low SHA values of acrylic-based resilient denture liners [14]. When they leach out and are partially replaced by water, these materials will harden, which can support further contamination with microorganisms [50]. For silicone resilient denture liners, it is suggested that mechanical properties depend on the density of crosslinks [51,52]. This aspect should be evaluated in further studies. 

A difference in how acrylic-based or silicone resilient denture liners are affected by the addition of CS salts was observed in this study. The modified silicone resilient denture liner mainly showed higher or similar SHA values compared to the control group after 30 days. However, the modified acrylic-based resilient denture liner showed lower SHA values compared to control group after 30 days, concluding that the addition of CS salts might slow down the hardening process of the acrylic-based resilient denture liner.

Changes in SHA when adding antimicrobial particles were also reported by other authors [20,21,43]. Chladek et al. modified a silicone resilient denture liner and found SHA values to decrease with increasing concentration of silver nanoparticles [21] Contrary, the present study showed SHA values of the modified silicone resilient denture liner increasing or showing similar values compared to control group. Urban et al. reported that the addition of antimicrobial agents to an acrylic-based resilient denture liner did result in higher SHA values compared to the control group after 14 days [20]. On the contrary, Bueno et al. reported significant decreases in SHA after 14 days for all modifications of an acrylic-based resilient denture liner with antifungals compared to the control group [43]. The tendency of the latter result was confirmed by the present study. Whilst after 14 days most modifications of the acrylic-based resilient denture liner showed SHA values lower than the control group, at 30 days all modifications were less hard than the control group. 

As surface roughness influences the adherence of microorganisms [12], it is a parameter to consider when researching and advancing resilient denture liners, e.g., receiving antimicrobial/antibiofilm properties by modification. Smooth surfaces should be regarded favourable, especially as resilient denture liners tend to have a more irregular surface than hard acrylic denture base materials [19,26] and are therefore more prone to biofilm accumulation [19,27]. In the present study, the acrylic-based resilient denture liner showed higher Ra values than the silicone resilient denture liner, which was also found by other authors [15], and therefore seems less favourable in terms of *C. albicans* adhesion.

The 0.2 µm threshold was mostly not achieved in this study. Other studies showed similar results for resilient denture liners as mean Ra values ≤0.2 µm did not occur for these materials and often much higher values were reached [20,53,54,55,56,57,58]. In a clinical study, Ra values of 1.0 µm for a silicone resilient denture liner and 1.2 µm for an acrylic-based resilient denture liner were found after a 21-day long wear [59]. It should be stressed that original studies postulating a Ra threshold value of 0.2 µm refer to hard materials [60,61,62]. In theory, this value should be considered ideal because of its link to bacterial adhesion. However, 0.2 µm does not seem to be a clinically achievable and maintainable Ra value for resilient denture liners [59]. It also should be considered that in the present study low Ra values might occur due to specimen preparation against glass plates, which does not coincide with the clinical protocol and is a limitation of the present study. Higher values might be expected when resilient denture liners polymerize in contact with patients’ mucosa. 

In the present study, all modified and unmodified silicone resilient denture liner specimens became rougher over the 30-day trial. In the case of the acrylic-based resilient denture liner, control group and all modifications with CS-HCl as well as with 0.1% and 0.2% CS-G became less rough over time or ended up with similar values at 24 h and 30 days. Similar effects for acrylic-based resilient denture liners were shown by Bueno et al. and Urban et al. [20,43].

Most of the times, the control group proved least rough in these studies as well as in the present one. In the present study, the majority of modified groups showed significant increases in Ra compared to the control group. Many modified groups tested by other authors showed no significant change in surface roughness compared to the control group [20,43]. The antimicrobial agents used by Bueno et al. or Urban et al. were different to the ones used in the present study and therefore cannot be compared directly.

In this study especially, UG-specimens developed small blisters, presumably where CS salt particles had swollen or dissolved in the distilled water. This was dependent on concentration and time. Higher roughness values with increased CS salt concentration and time seem consequential. Presumably, CS salts either expanded by taking up water or were rinsed out leaving small blisters on the surface. Further tests to clarify this aspect are needed. Dissolving of a drug incorporated in a methacrylate polymer matrix when in contact with water has been described before though this was accepted for drug loading purposes [63]. If CS salt particles do get washed out, this might affect the antimicrobial properties and therefore should be considered in future microbial testing. As antimicrobial activities for CS salts against fungi, Gram-positive and Gram-negative bacteria have been reported [31,38,39,64], CS-HCl and CS-G are suitable materials for modifications of resilient denture liners.

Further limitations of this study include that only two material properties were examined. Sorption, solubility, bond strength, wettability and resistance to colour changes need to be explored in further studies. Moreover, many clinical aspects could not be included in the in vitro methodology and need further investigation. 

Although roughness was affected negatively in this study, hardness of the specimens was satisfying. Therefore, the concept can be considered acceptable for developing materials with antimicrobial/antibiofilm activity.

In vitro studies testing other material properties such as bonding properties to base materials, sorption, solubility, etc., and especially antifungal tests should follow up to further investigate the properties of CS salts when mixed into resilient denture liners. In the case of antimicrobial properties, positive results have been reported for nano-chitosan particles in light-cured resins [65] as well as CS and CS oligosaccharides [40], CS nanoparticles [42] and CS and quaternized CS in tissue conditioners [41]. In contrast, modification of hard denture base materials by adding CS-HCl or CS-G showed unsatisfactory antifungal/antibiofilm activity (own unpublished data). However, modification of resilient denture liners could still promote antifungal activity despite increasing roughness, as the roughness is an unfavourable property from the beginning, promoting microorganisms’ adhesion and as soft materials are not directly comparable with hard denture materials.

## 5. Conclusions

Within the limitations of this study, modifying resilient denture liners with CS salts did not change the hardness of the material in a relevant range. However, the modification seems to be unfavourable concerning surface roughness. Further studies, especially antifungal tests, should be conducted before a final clinical assessment of resilient denture liners modified with CS salts is possible.

## Figures and Tables

**Table 1:** Descriptive data for Shore A hardness.

	Median T1	Min T1	Max T1	Median T2	Min T2	Max T2	Median T3	Min T3	Max T3	Median T4	Min T4	Max T4
UG-CS-HCl-0.1	29.6	29.2	29.7	34.6^aA^	33.8	35.4	36.2^aA^	36.0	36.5	35.0	34.7	35.4
UG-CS-HCl-0.2	29.5	29.1	29.7	35.2^aA^	33.3	35.5	36.4^aA^	35.8	36.7	35.4^aA^	35.1	35.8
UG-CS-HCl-0.4	30.1^aA^	29.4	30.4	35.6^aA^	35.0	36.1	36.7^aA^	35.6	36.9	35.4^aA^	35.0	36.0
UG-CS-HCl-1	30.9^aA^	30.2	31.1	35.9^aA^	35.4	36.2	36.5^aA^	35.3	36.7	35.2	34.7	35.6
UG-CS-G-0.1	29.8	29.2	30.0	34.9^aA^	34.6	35.8	36.3^aA^	35.5	36.4	35.9^aA^	35.3	36.2
UG-CS-G-0.2	30.2^aA^	29.6	30.7	36.0^aA^	35.5	36.3	36.8^aA^	36.6	37.0	36.2^aA^	35.7	36.5
UG-CS-G-0.4	30.1^aA^	29.6	30.4	36.0^aA^	35.2	36.2	36.5^aA^	35.7	36.7	35.8^aA^	35.4	36.0
UG-CS-G-1	30.6^aA^	30.4	31.1	36.0^aA^	35.7	36.1	36.4^aA^	35.4	36.8	35.5^aA^	34.9	36.4
UG-C	29.6	29.0	29.7	34.1	32.7	34.8	35.1	33.8	35.4	35.1	34.6	35.3
COE-CS-HCl-0.1	11.9	9.6	13.7	15.9	13.4	17.9	17.5	15.4	18.9	18.6	16.9	21.0
COE-CS-HCl-0.2	11.4	10.4	12.9	15.0	13.5	16.4	16.6	15.4	19.8	16.3^aA^	14.9	19.0
COE-CS-HCl-0.4	10.4	9.0	12.0	14.1	12.8	15.0	14.7^aA^	13.2	16.3	16.6^aA^	15.0	17.8
COE-CS-HCl-1	11.7	9.8	12.5	14.8	12.4	16.0	14.9^aA^	12.3	16.4	15.7^aA^	14.4	17.0
COE-CS-G-0.1	11.8	9.6	12.5	16.2	14.0	17.6	17.3	14.5	18.3	19.1	15.9	19.9
COE-CS-G-0.2	10.4	9.3	11.7	14.6	12.8	16.0	15.9^a^	14.5	17.8	17.3^aA^	15.9	18.8
COE-CS-G-0.4	10.9	10.3	13.1	15.0	13.3	16.9	17.7	15.0	18.5	17.6^aA^	16.3	20.2
COE-CS-G-1	10.9	9.2	13.4	14.6	12.6	16.5	16.0	13.6	17.5	17.4^aA^	15.5	19.3
COE-C	11.2	8.5	13.3	15.2	10.9	17.8	17.1	13.6	18.6	19.8	16.8	21.6

Ufi Gel P (UG), Coe-Soft (COE), chitosan hydrochloride (CS-HCl), chitosan glutamate (CS-G), control group (C), concentration in % w/w of CS salt added (0.1, 0.2, 0.4, 1), Shore A hardness (SHA), 24 h after specimen preparation (T1), 7 d after specimen preparation (T2), 14 d after specimen preparation (T3), 30 d after specimen preparation (T4), minimum (min), maximum (max), a superscript letter indicates significant difference to respective control (*p*-values raw ≤ 0.05), A superscript letter indicates significant difference to respective control (*p*-values adjusted ≤ 0.05).

**Table 2:** Shore A hardness *p*-values (≤0.05) raw and adjusted (adj.) by Bonferroni–Holm correction for Ufi Gel P.

	*P* Raw	*P* Adj.	*P* Raw	*P* Adj.	*P* Raw	*P* Adj.	*P* Raw	*P* Adj.
	SHA T1	SHA T1	SHA T2	SHA T2	SHA T3	SHA T3	SHA T4	SHA T4
UG-CS-HCl-0.1/UG-C	n.s.	n.s.	0.015	0.015	<0.001	<0.001	n.s.	n.s.
UG-CS-HCl-0.2/UG-C	n.s.	n.s.	0.005	0.01	<0.001	<0.001	0.002	0.008
UG-CS-HCl-0.4/UG-C	0.001	0.003	<0.001	<0.001	<0.001	<0.001	0.004	0.012
UG-CS-HCl-1/UG-C	<0.001	<0.001	<0.001	<0.001	<0.001	<0.001	n.s.	n.s.
UG-CS-G-0.1/UG-C	n.s.	n.s.	<0.001	<0.001	<0.001	<0.001	<0.001	<0.001
UG-CS-G-0.2/UG-C	<0.001	<0.001	<0.001	<0.001	<0.001	<0.001	<0.001	<0.001
UG-CS-G-0.4/UG-C	<0.001	<0.001	<0.001	<0.001	<0.001	<0.001	<0.001	<0.001
UG-CS-G-1/UG-C	<0.001	<0.001	<0.001	<0.001	<0.001	<0.001	0.007	0.007

Ufi Gel P (UG), chitosan hydrochloride (CS-HCl), chitosan glutamate (CS-G), control group (C), concentration in % w/w of CS salt added (0.1, 0.2, 0.4, 1), n.s. non-significant, Shore A hardness (SHA), 24 h after specimen preparation (T1), 7 d after specimen preparation (T2), 14 d after specimen preparation (T3), 30 d after specimen preparation (T4).

**Table 3:** Shore A hardness *p*-values (≤0.05) raw and adjusted (adj.) by Bonferroni-Holm correction for Coe-Soft.

	*P* Raw	*P* Adj.	*P* Raw	*P* Adj.	*P* Raw	*P* Adj.	*P* Raw	*P* Adj.
	SHA T1	SHA T1	SHA T2	SHA T2	SHA T3	SHA T3	SHA T4	SHA T4
COE-CS-HCl-0.1/COE-C	n.s.	n.s.	n.s.	n.s.	n.s.	n.s.	n.s.	n.s.
COE-CS-HCl-0.2/COE-C	n.s.	n.s.	n.s.	n.s.	n.s.	n.s.	<0.001	<0.001
COE-CS-HCl-0.4/COE-C	n.s.	n.s.	n.s.	n.s.	0.001	0.004	<0.001	<0.001
COE-CS-HCl-1/COE-C	n.s.	n.s.	n.s.	n.s.	0.002	0.006	<0.001	<0.001
COE-CS-G-0.1/COE-C	n.s.	n.s.	n.s.	n.s.	n.s.	n.s.	n.s.	n.s.
COE-CS-G-0.2/COE-C	n.s.	n.s.	n.s.	n.s.	0.043	n.s.	0.001	0.004
COE-CS-G-0.4/COE-C	n.s.	n.s.	n.s.	n.s.	n.s.	n.s.	0.011	0.022
COE-CS-G-1/COE-C	n.s.	n.s.	n.s.	n.s.	n.s.	n.s.	0.002	0.006

Coe-Soft (COE), chitosan hydrochloride (CS-HCl), chitosan glutamate (CS-G), control group (C), concentration in % w/w of CS salt added (0.1, 0.2, 0.4, 1), n.s. non-significant, Shore A hardness (SHA), 24 h after specimen preparation (T1), 7 d after specimen preparation (T2), 14 d after specimen preparation (T3), 30 d after specimen preparation (T4).

**Table 4:** Descriptive data for surface roughness (Ra) in µm.

	Median T1	Min T1	Max T1	Median T2	Min T2	Max T2	Median T3	Min T3	Max T3	Median T4	Min T4	Max T4
UG-CS-HCl-0.1	0.38^aA^	0.30	0.50	0.65^aA^	0.42	0.77	0.58^aA^	0.37	1.25	0.69^aA^	0.57	0.89
UG-CS-HCl-0.2	0.54^aA^	0.33	0.83	0.66^aA^	0.49	0.78	0.91^aA^	0.56	1.30	1.04^aA^	0.70	1.46
UG-CS-HCl-0.4	0.78^aA^	0.50	1.32	1.29^aA^	0.64	2.70	1.67^aA^	1.21	1.94	1.82^aA^	0.99	2.55
UG-CS-HCl-1	0.99^aA^	0.70	1.34	2.42^aA^	1.41	3.39	3.11^aA^	1.48	6.07	3.32^aA^	2.24	4.87
UG-CS-G-0.1	0.50^aA^	0.32	0.87	0.71^aA^	0.43	0.91	0.68^aA^	0.52	1.14	0.65^aA^	0.44	1.18
UG-CS-G-0.2	0.68^aA^	0.42	0.85	0.87^aA^	0.67	1.15	0.95^aA^	0.68	1.25	1.00^aA^	0.69	1.30
UG-CS-G-0.4	0.85^aA^	0.64	1.20	1.16^aA^	0.96	2.04	1.33^aA^	1.05	2.03	1.31^aA^	1.10	1.59
UG-CS-G-1	0.96^aA^	0.71	1.23	1.79^aA^	1.23	2.48	2.27^aA^	1.48	2.96	2.37^aA^	1.58	3.09
UG-C	0.20	0.16	0.28	0.31	0.23	0.47	0.30	0.19	0.34	0.30	0.20	0.43
COE-CS-HCl-0.1	5.18^aA^	4.54	10.40	5.00^aA^	4.04	5.89	4.11^aA^	3.16	5.96	3.74^aA^	3.32	4.53
COE-CS-HCl-0.2	4.69^aA^	4.45	5.54	4.47^aA^	4.11	4.99	4.14^aA^	3.62	4.56	3.93^aA^	3.55	4.35
COE-CS-HCl-0.4	5.14^aA^	4.37	7.22	4.91^aA^	4.01	6.43	4.65^aA^	4.15	6.95	4.74^aA^	4.26	6.84
COE-CS-HCl-1	5.05^aA^	4.52	5.80	4.97^aA^	3.81	5.37	4.69^aA^	3.69	5.62	5.06^aA^	3.93	5.87
COE-CS-G-0.1	4.61	4.13	5.36	4.26^aA^	3.73	5.01	4.00^aA^	3.61	4.67	4.11^aA^	3.13	5.09
COE-CS-G-0.2	4.99^aA^	4.33	5.79	4.33^aA^	3.67	5.33	4.15^aA^	3.29	4.95	3.87^aA^	3.57	4.51
COE-CS-G-0.4	4.73^aA^	4.05	5.66	4.84^aA^	4.05	5.43	4.90^aA^	4.21	5.46	4.89^aA^	4.44	6.01
COE-CS-G-1	5.03^aA^	4.60	5.91	5.58^aA^	4.73	5.79	5.53^aA^	5.13	6.15	5.41^aA^	4.70	6.28
COE-C	4.25	3.48	4.71	3.71	3.23	4.25	3.38	2.92	3.88	3.06	2.65	3.32

Ufi Gel P (UG), Coe-Soft (COE), chitosan hydrochloride (CS-HCl), chitosan glutamate (CS-G), control group (C), concentration in % w/w of CS salt added (0.1, 0.2, 0.4, 1), 24 h after specimen preparation (T1), 7 d after specimen preparation (T2), 14 d after specimen preparation (T3), 30 d after specimen preparation (T4), minimum (min), maximum (max), a superscript letter indicates significant difference to respective control (*p*-values raw ≤ 0.05), A superscript letter indicates significant difference to respective control (*p*-values adjusted ≤ 0.05).

**Table 5:** Surface roughness *p*-values (≤0.05) raw and adjusted (adj.) by Bonferroni-Holm correction for Coe-Soft.

	*P* Raw	*P* Adj.	*P* Raw	*P* Adj.	*P* Raw	*P* Adj.	*P* Raw	*P* Adj.
	Ra T1	Ra T1	Ra T2	Ra T2	Ra T3	Ra T3	Ra T4	Ra T4
COE-CS-HCl-0.1/COE-C	<0.001	<0.001	<0.001	<0.001	0.003	0.003	<0.001	<0.001
COE-CS-HCl-0.2/COE-C	0.001	0.001	<0.001	<0.001	<0.001	<0.001	<0.001	<0.001
COE-CS-HCl-0.4/COE-C	<0.001	<0.001	<0.001	<0.001	<0.001	<0.001	<0.001	<0.001
COE-CS-HCl-1/COE-C	<0.001	<0.001	<0.001	<0.001	<0.001	<0.001	<0.001	<0.001
COE-CS-G-0.1/COE-C	n.s.	n.s.	0.009	0.009	<0.001	<0.001	<0.001	<0.001
COE-CS-G-0.2/COE-C	0.002	0.006	0.001	0.002	0.004	0.004	<0.001	<0.001
COE-CS-G-0.4/COE-C	0.011	0.022	<0.001	<0.001	<0.001	<0.001	<0.001	<0.001
COE-CS-G-1/COE-C	<0.001	<0.001	<0.001	<0.001	<0.001	<0.001	<0.001	<0.001

Coe-Soft (COE), chitosan hydrochloride (CS-HCl), chitosan glutamate (CS-G), control group (C), concentration in % w/w of CS salt added (0.1, 0.2, 0.4, 1), n.s. non-significant, surface roughness (Ra), 24 h after specimen preparation (T1), 7 d after specimen preparation (T2), 14 d after specimen preparation (T3), 30 d after specimen preparation (T4).

## Appendix A

**Table A1:** CS salts specifications given by the manufacturer.

CS Salt	Product	Manufacturer	Product No.	Batch No.	Specification	Limit	Results
CS-HCl	Chitoceuticals Chitosan HCl	Heppe Medical Chitosan GmbH	44001	312-180116-02	DDA (%)	80–95	84
					MW (kDA)	30–400	48
					Viscosity (1% in water, mPas)	2–200	30
					pH (1% in water, 20 °C)	4.0–6.0	4
CS-G	Chitoceuticals Chitosan Glutamate	Heppe Medical Chitosan GmbH	44006	312-270116-02	DDA (%)	≥80	98
					MW (kDA)	30–600	228
					Viscosity (1% in water, mPas)	≥10	486
					pH (1% in water, 20 °C)	3.0–7.0	5

Chitosan (CS), chitosan-hydrochloride (CS-HCl), chitosan glutamate (CS-G), number (No.), molecular weight (MW), degree of deacetylation (DDA).

**Table A2:** Mean values and standard deviation (SD) for Shore A hardness.

	Mean T1	SD T1	Mean T2	SD T2	Mean T3	SD T3	Mean T4	SD T4
UG-CS-HCl-0.1	29.5	0.16	34.6	0.45	36.3	0.16	35.0	0.23
UG-CS-HCl-0.2	29.5	0.19	34.9	0.69	36.4	0.25	35.4	0.21
UG-CS-HCl-0.4	30.0	0.29	35.6	0.38	36.5	0.41	35.5	0.35
UG-CS-HCl-1	30.8	0.25	35.8	0.24	36.4	0.43	35.2	0.28
UG-CS-G-0.1	29.7	0.25	35.1	0.50	36.1	0.32	35.9	0.25
UG-CS-G-0.2	30.2	0.32	35.9	0.27	36.8	0.12	36.1	0.27
UG-CS-G-0.4	30.1	0.28	35.9	0.31	36.3	0.38	35.8	0.20
UG-CS-G-1	30.7	0.23	35.9	0.13	36.3	0.39	35.5	0.45
UG-C	29.5	0.23	34.0	0.65	35.0	0.45	35.0	0.21
COE-CS-HCl-0.1	11.9	1.29	15.7	1.52	17.4	1.06	18.6	1.31
COE-CS-HCl-0.2	11.5	0.87	14.9	1.04	16.9	1.39	16.7	1.41
COE-CS-HCl-0.4	10.6	0.89	14.0	0.72	14.7	1.12	16.5	1.04
COE-CS-HCl-1	11.3	0.93	14.7	0.97	14.8	1.10	15.7	0.84
COE-CS-G-0.1	11.4	0.90	15.8	1.11	17.3	1.11	18.6	1.27
COE-CS-G-0.2	10.3	0.83	14.6	1.04	15.9	1.06	17.5	0.97
COE-CS-G-0.4	11.3	1.05	15.1	1.22	17.2	1.19	17.8	1.22
COE-CS-G-1	10.8	1.31	14.6	1.19	15.7	1.37	17.3	1.45
COE-C	11.3	1.35	15.0	1.87	16.9	1.42	19.6	1.31

Ufi Gel P (UG), Coe-Soft (COE), chitosan-hydrochloride (CS-HCl), chitosan glutamate (CS-G), control group (C), concentration in % w/w of CS salt added (0.1, 0.2, 0.4, 1), 24 h after specimen preparation (T1), 7 d after specimen preparation (T2), 14 d after specimen preparation (T3), 30 d after specimen preparation (T4).

**Table A3:** Mean values and standard deviation (SD) for surface roughness in µm.

	Mean T1	SD T1	Mean T2	SD T2	Mean T3	SD T3	Mean T4	SD T4
UG-CS-HCl-0.1	0.40	0.071	0.64	0.102	0.62	0.253	0.72	0.105
UG-CS-HCl-0.2	0.55	0.143	0.65	0.096	0.96	0.229	1.05	0.269
UG-CS-HCl-0.4	0.80	0.249	1.37	0.572	1.65	0.238	1.70	0.577
UG-CS-HCl-1	1.04	0.211	2.45	0.640	3.31	1.311	3.35	0.910
UG-CS-G-0.1	0.51	0.160	0.68	0.158	0.75	0.198	0.72	0.219
UG-CS-G-0.2	0.66	0.141	0.89	0.140	0.95	0.218	0.99	0.161
UG-CS-G-0.4	0.87	0166	1.23	0.326	1.41	0.342	1.34	0.155
UG-CS-G-1	0.97	0.166	1.83	0.368	2.13	0.471	2.32	0.570
UG-C	0.21	0.036	0.32	0.086	0.29	0.043	0.31	0.072
COE-CS-HCl-0.1	5.74	1.707	5.00	0.628	4.20	0.746	3.91	0.425
COE-CS-HCl-0.2	4.83	0.373	4.50	0.244	4.13	0.293	3.99	0.283
COE-CS-HCl-0.4	5.23	0.782	4.90	0.652	4.92	0.823	4.85	0.747
COE-CS-HCl-1	5.06	0.414	4.83	0.453	4.79	0.686	5.09	0.584
COE-CS-G-0.1	4.65	0.446	4.32	0.396	4.03	0.359	4.09	0.591
COE-CS-G-0.2	4.92	0.479	4.38	0.451	4.11	0.548	3.94	0.297
COE-CS-G-0.4	4.79	0.488	4.74	0.388	4.84	0.415	4.95	0.435
COE-CS-G-1	5.04	0.440	5.32	0.455	5.51	0.345	5.44	0.500
COE-C	4.22	0.352	3.75	0.356	3.40	0.286	3.09	0.194

Ufi Gel P (UG), Coe-Soft (COE), chitosan-hydrochloride (CS-HCl), chitosan glutamate (CS-G), control group (C), concentration in % w/w of CS salt added (0.1, 0.2, 0.4, 1), 24 h after specimen preparation (T1), 7 d after specimen preparation (T2), 14 d after specimen preparation (T3), 30 d after specimen preparation (T4).
